# mTOR signaling: implications for cancer and anticancer therapy

**DOI:** 10.1038/sj.bjc.6602902

**Published:** 2005-12-13

**Authors:** E Petroulakis, Y Mamane, O Le Bacquer, D Shahbazian, N Sonenberg

**Affiliations:** 1 Department of Biochemistry, McGill Cancer Centre, McGill University, 3655 Promenade Sir William Osler, Montreal, QUE, Canada H3G 1Y6

**Keywords:** translational control, eIF4F, eIF4E binding proteins, rapamycin, mTOR, malignant transformation

## Abstract

Mounting evidence links deregulated protein synthesis to tumorigenesis via the translation initiation factor complex eIF4F. Components of this complex are often overexpressed in a large number of cancers and promote malignant transformation in experimental systems. mTOR affects the activity of the eIF4F complex by phosphorylating repressors of the eIF4F complex, the eIF4E binding proteins. The immunosuppressant rapamycin specifically inhibits mTOR activity and retards cancer growth. Importantly, mutations in upstream negative regulators of mTOR cause hamartomas, haemangiomas, and cancers that are sensitive to rapamycin treatment. Such mutations lead to increased eIF4F formation and consequently to enhanced translation initiation and cell growth. Thus, inhibition of translation initiation through targeting the mTOR-signalling pathway is emerging as a promising therapeutic option.

## 

Rapamycin (Rapamune®, Wyeth Ayerst) is a specific inhibitor of the target of rapamycin (TOR) (for review see Hay and Sonenberg, 2004). The protein kinase TOR genes (TOR1 and TOR2) were first identified in the early 1990s in a screen for rapamycin-resistant yeast mutants (Heitman *et al*, 1991). The mammalian orthologue (mTOR), is also referred to as FKBP12-rapamycin associated protein (FRAP), rapamycin and FKBP12 target (RAFT), rapamycin target (RAPT), or sirolimus effector protein (SEP). Rapamycin forms a complex with the immunophilin FK506 binding protein-12 (FKBP12), which binds to the FKBP12-rapamycin binding (FRB) domain of mTOR and inhibits its kinase activity. Rapamycin inhibits the growth of a broad spectrum of cancers including rhabdomyosarcoma, neuroblastoma, glioblastoma, small cell lung carcinoma, osteosarcoma, pancreatic cancer, leukaemia, B-cell lymphoma, and breast and colon cancer-derived cells (Huang and Houghton, 2002). The rapamycin analogues, CCI-779 (Wyeth-Ayerst, PA, USA), RAD001 (Novartis, Switzerland), and AP23573 (Ariad Pharmaceuticals, MA, USA) have shown promise in clinical trials (Panwalkar *et al*, 2004).

mTOR functions by integrating extracellular signals (growth factors and hormones), with amino-acid availability and intracellular energy status to control translation rates and additional metabolic processes (Hay and Sonenberg, 2004). mTOR enhances translation initiation in part by phosphorylating two major targets, the eIF4E binding proteins (4E-BPs) and the ribosomal protein S6 kinases (S6K1 and S6K2) that cooperate to regulate translation initiation rates.

### 

#### Translation initiation is implicated in tumorigenesis

All nuclear transcribed eukaryotic mRNAs contain a ‘cap’ structure, m^7^GpppN (where ‘N’ is any nucleotide and ‘m’ is a methyl group), at the 5′ terminus. The ‘cap’ is specifically bound by the initiation factor eIF4E that associates with two additional initiation factors, eIF4G (a large scaffolding protein) and eIF4A (a helicase which is believed to unwind mRNA 5′ secondary structures), to form the eIF4F complex, which facilitates the recruitment of ribosomes to the mRNA (Gingras *et al*, 1999).

Several studies demonstrated that eIF4E acts as an oncogene (for a recent review see Mamane *et al*, 2004). eIF4E levels are limiting for cap-dependent translation in most systems and overexpression of eIF4E in rodent cells causes malignant transformation (Lazaris-Karatzas *et al*, 1990; Mamane *et al*, 2004). Therefore, increased eIF4E expression in cancer cells is thought to enhance eIF4F complex formation and as a consequence, the translation of a subset of mRNAs that contain highly structured 5′ untranslated regions (UTRs), such as vascular endothelial growth factor (VEGF) and ornithine decarboxylase (ODC). Indeed, eIF4E is elevated in numerous types of cancers, including bronchioalveolar, bladder, head and neck, liver, colon, and breast cancers (De Benedetti and Graff, 2004). Consistent with this, two important studies demonstrated that overexpression of eIF4E in mice promotes lymphomagenesis (Ruggero *et al*, 2004; Wendel *et al*, 2004). Furthermore, the importance of eIF4E's role in tumorigenesis is reinforced by the finding that eIF4F complex is necessary for maintaining tumour cell growth (Avdulov *et al*, 2004). Importantly, siRNA treatment to reduce eIF4E expression inhibits the growth of several cell lines including those of head and neck squamous carcinoma cells (Oridate *et al*, 2005).

Other components of the eIF4F complex are also implicated in malignant transformation. eIF4GI can transform NIH 3T3 cells, and is overexpressed in squamous cell lung carcinomas and breast cancer cell lines (see Mamane *et al* (2004)). eIF4A is also overexpressed in human melanoma cell lines and in primary hepatocellular carcinomas (see Mamane *et al* (2004)). Hence, these findings reinforce a molecular model where increased eIF4F complex promotes tumorigenesis.

#### Rapamycin inhibits cap-dependent translation

Growth inhibition by rapamycin is thought to be partially mediated by the inhibition of cap-dependent translation (Hay and Sonenberg, 2004). The mammalian 4E-BPs constitute a three-member family of translational inhibitors that bind to eIF4E, inhibit formation of the eIF4F complex and thus cap-dependent translation (Gingras *et al*, 1999). The 4E-BPs are dephosphorylated as a consequence of rapamycin treatment. Binding of 4E-BP1 (the best studied 4E-BP family member) to eIF4E is regulated by its phosphorylation status: the hypophosphorylated form (residues Thr37 and 46) of 4E-BP1 binds very tightly to eIF4E and inhibits cap-dependent translation. In response to stimuli such as growth factors, hormones, nutrients, or increased energy levels, mTOR phosphorylates 4E-BP1 primarily at residues Ser65 and Thr73. As a result, eIF4E is released from 4E-BP1 with a subsequent increase in cap-dependent translation (Mamane *et al*, 2004).

Several studies addressed the role of the 4E-BPs in tumour suppression. For example, 4E-BP overexpression counteracts eIF4E-, src-, or ras-mediated cellular transformation (Mamane *et al*, 2004). Also, a nonphosphorylatable mutant of 4E-BP1, which constitutively binds eIF4E, inhibits breast cancer cell growth more strongly than wild-type 4E-BP1 (Avdulov *et al*, 2004). In addition, the 4E-BPs appear to mediate rapamycin-sensitivity since rhabdomyosarcoma cells expressing very low levels of 4E-BP1 are refractory to growth inhibition by rapamycin (Dilling *et al*, 2002). Thus, the 4E-BPs are considered to be important mTOR targets that modulate cancer cell growth through a mechanism that involves cap-dependent translation.

#### Ribosomal protein S6 kinase

The ribosomal protein S6 kinases are also important substrates of mTOR. S6K1 (p70S6K; the better characterised S6 kinase) is implicated in the positive regulation of cell growth and proliferation (Fumagalli and Thomas, 2000). Interestingly, S6K1 activation correlates with enhanced translation of a subset of mRNAs that contain a terminal 5′ oligopyrimidine tract (TOP mRNAs) (Fumagalli and Thomas, 2000). These mRNAs encode ribosomal proteins, elongation factors, the poly-A binding protein and other components of the translational machinery that become selectively translated in response to growth factors. However, TOP mRNA translation remains intact even when both S6K1 and S6K2 genes are genetically disrupted in mice (Pende *et al*, 2004), indicating that S6Ks are not essential for the regulation of TOP mRNA translation.

S6K regulates mTOR through a negative feedback signalling pathway that affects insulin receptor substrate-1 (IRS-1) (Figure 1). S6K was shown to directly phosphorylate IRS-1 to inhibit phospohatidylinositol-3-kinase (PI3K) and Akt activation (Harrington *et al*, 2004). S6K activation decreases IRS-1 expression, while rapamycin treatment restores IRS-1 expression (Harrington *et al*, 2004). Also, deletion of S6K1 in mice renders them resistant to age- and high-fat diet-induced obesity while enhancing insulin sensitivity (Um *et al*, 2004). Lastly, Akt activation in response to insulin is enhanced in S6K-null mice, thus implicating S6K1 in diabetes and obesity (Um *et al*, 2004).

#### Other translational targets of mTOR

Other rapamycin-sensitive targets involved in translational regulation exist. The translation initiation factor eIF4B, which stimulates the activity of eIF4A in unwinding duplex RNA, is phosphorylated by S6K1 on serine 422 (Raught *et al*, 2004). mTOR also mediates the phosphorylation of serines 1108, 1148, and 1192 in the C-terminus of eIF4G (Raught *et al*, 2000). In addition, mTOR regulates the elongation stage of protein synthesis by modulating elongation factor (eEF2) kinase (Proud, 2004).

#### PI3K/Akt/mTOR signaling

Growth factors, mitogens and hormones activate the PI3K signaling pathway and consequently mTOR (Hay and Sonenberg, 2004) (Figure 1). Nutrients (amino acids, glucose) also regulate mTOR activity to affect translation (Proud, 2004). PI3K phosphorylates phosphatidylinositol-4,5-bisphosphate (PIP2) to generate phosphatidylinositol-3,4,5-triphosphate (PIP3). PIP3 binds and activates pleckstrin homology (PH) domain-bearing proteins such as Akt and the phosphoinositide dependent kinase (PDK) family members. PDK1 and the Ser/Thr kinase Akt are recruited to the membrane where PDK1 phosphorylates and activates Akt. In turn, Akt phosphorylates the tuberous sclerosis complex, TSC1/TSC2 (hamartin/tuberin) that serves as a GTPase activating protein (GAP) for the small G protein, Ras homolog enriched in brain (Rheb). Rheb, in its GTP-bound state, can activate mTOR.

## 

### Negative regulators of mTOR are implicated in tumour suppression

#### PTEN

PTEN is a lipid phosphatase that acts on the lipid substrate PIP3 to convert it to PIP2. PIP3 accumulation results from PTEN-inactivating mutations to effect cell size, adhesion, motility, and angiogenesis (Inoki *et al*, 2005a). PTEN is frequently mutated in many cancers and in a group of cancer-like syndromes including Cowden, Lhermitte–Duclos, Bannayan–Zonana, and Proteus syndromes that are characterised by the emergence of hamartomas (Inoki *et al*, 2005a). A critical outcome of PTEN inactivation is an increase in mTOR activity, resulting in the phosphorylation of 4E-BPs and S6Ks. Strikingly, PTEN−/− cells are hypersensitive to growth inhibition by rapamycin and CCI-779 (Guertin and Sabatini, 2005).

#### AMP-activated protein kinase (*AMPK*)

mTOR activity becomes repressed under conditions of energy deprivation, in which an elevated AMP/ATP intracellular ratio causes activation of AMPK. Stressful conditions, such as nutrient deprivation, hypoxia, heat shock, and ischaemia diminish cellular energy reserves. The link between mTOR inhibition and AMPK was first demonstrated with the use of AICAR (5′-phosphoribosyl-5-aminoimidazole-4-carboxamide ribonucleoside), an AMPK activator, thus linking the amino acid- and energy-sensing functions of mTOR. Metformin, an antidiabetic drug, also activates AMPK and is hypothesised to reduce the risk of cancer in patients with type 2 diabetes (Evans *et al*, 2005). Thus, targeting AMPK may be an interesting therapeutic option for cancer therapy.

#### LKB1

LKB1 is a serine/threonine kinase that phosphorylates and activates AMPK. LKB1 is frequently mutated in Peutz-Jeghers syndrome (PJS), an autosomal dominant inherited cancer predisposing patients to the development of tumours mainly throughout the gastrointestinal tract. Disruption of LKB1 in mice causes gastrointestinal and hepatic polyps (Bardeesy *et al*, 2002). In LKB1-deficient cells, phosphorylation AMPK is defective and cells resist mTOR inactivation in response to AICAR (Shaw *et al*, 2004). Importantly, 4E-BP1 is less enriched in eIF4E-bound cap analogue precipitates from LKB1-deficient cells (Shaw *et al*, 2004). This is indicative of enhanced eIF4F formation as a result of increased mTOR signaling caused by LKB1-deficiency. LKB1-mediated inhibition of mTOR also involves the TSC1/2 complex, thus demonstrating the link between LKB1 and mTOR through the regulation of AMPK and TSC1/2 activities (Shaw *et al*, 2004).

#### TSC1/TSC2

TSC1 and TSC2 are mutated in tuberous sclerosis complex (TSC) patients. In response to low energy levels, TSC2 becomes phosphorylated by AMPK, resulting in increased stability of the TSC1/2 complex. The stabilised TSC1/2 complex inhibits mTOR and protects cells from energy deprivation-induced apoptosis (see recent reviews by (Inoki *et al*, 2005b; Kwiatkowski and Manning, 2005)). TSC2 is also phosphorylated by Akt leading to its inactivation and consequently to increased mTOR activity. Furthermore, S6K phosphorylation is elevated in TSC−/− cells and is rapidly abrogated by rapamycin treatment. Activation of the extracellular signal-regulated kinase (Erk) by the Ras signaling pathway also leads to TSC1/2 inactivation through phosphorylation of TSC2 on Ser664 (Ma *et al*, 2005). Taken together, the TSC1/2 complex functions as a key player in the regulation of the mTOR pathway by receiving inputs from PI3K/PTEN/Akt and Ras signalling pathways, energy levels, and amino acids to regulate translation initiation and affect cell growth and proliferation.

## MTOR COMPLEXES REGULATE RAPAMYCIN SENSITIVITY

Recent findings shed new light on the mechanisms of mTOR sensitivity to rapamycin. Three key interacting partners of mTOR (raptor, G*β*L, and rictor) modulate many of mTOR's rapamycin-sensitive and insensitive functions. These evolutionarily conserved proteins have been characterised in yeast and mammalian cells. Raptor is the mammalian ortholog of yeast Kog1 (Kontroller of growth 1) (reviewed in Kim and Sabatini (2004), Lorberg and Hall (2004)). Raptor interacts with mTOR and tethers it to its downstream effectors through a TOS (TOR signaling) motif found in the mTOR substrates, 4E-BP1 and S6K1 (amino acid sequences: FEMDI and FDIDL, respectively) (reviewed in Proud (2004)). The raptor/mTOR interaction is nutrient-sensitive and is dependent on the presence of another partner, G*β*L (a mammalian ortholog of yeast Lst8p) (Guertin and Sabatini, 2005). G*β*L/mLst8 interacts with the mTOR kinase domain independently of raptor and potentiates mTOR activity. Rictor (rapamycin insensitive component of TOR; the yeast AVO3 ortholog), is involved in the control of cytoskeleton organisation (Lorberg and Hall, 2004; Guertin and Sabatini, 2005). Unlike raptor/G*β*L/mTOR (mTORC1 complex), the rictor/mTOR (mTORC2 complex) interaction is insensitive to rapamycin treatment. Most importantly, the rictor/mTOR complex is required for Akt phosphorylation on Ser473 to achieve its full activation (Guertin and Sabatini, 2005). Although the activity of the rictor/mTOR complex is insensitive to short periods of rapamycin treatment, it has been suggested that long-term exposure to rapamycin could prevent newly-synthesised mTOR molecules from associating with rictor, thereby preventing rictor/mTOR-mediated Akt phosphorylation (Guertin and Sabatini, 2005). This model predicts that rictor could serve as a new therapeutic target in mTOR-linked hamartoma syndromes (such as tuberous sclerosis) and PTEN-mutated cancers, where rapamycin therapy has already proven beneficial.

## RAPAMYCIN IN COMBINATION THERAPY

In Peutz Jeghers syndrome, Tuberous Sclerosis, and other diseases where PTEN is inactivated, the use of rapamycin as a clinical means to reverse the effect of elevated mTOR activity is an attractive option (Inoki *et al*, 2005a). These diseases are distinct from other hamartoma-associated disorders (such as VHL syndrome) since they have an established molecular link to mTOR (Inoki *et al*, 2005a). Earlier studies demonstrated that PTEN-inactivated tumour cells exhibit enhanced sensitivity to the rapamycin analog CCI-779 (Guertin and Sabatini, 2005). More recently, several studies have shown that rapamycin treatment, in combination with other chemotherapeutic drugs, can lead to enhanced selective killing of cancer cells. In particular, the protein tyrosine kinase (PTK) inhibitor, Imatinib (Gleevec, STI571) synergises with rapamycin to inhibit BCR/ABL transformed cells (Mohi *et al*, 2004). The effect of rapamycin may be enhanced as a result of Imatinib-induced Akt/mTOR signaling, a complication that is thought to lead to Imatinib resistance (Burchert *et al*, 2005). Rapamycin can also synergise with paclitaxel, carboplatin, and vinorelbine to induce apoptosis in breast cancer cells (Mondesire *et al*, 2004). Cisplatin-induced apoptosis of A549 lung cancer cells is also significantly enhanced when combined with RAD001 (Beuvink *et al*, 2005). This could be in part due to reduced translation of p53-activated p21 mRNA in A549 and MCF7 cells treated with RAD001, thereby allowing the dosage of cisplatin to be reduced (Beuvink *et al*, 2005). Also, the use of the EGFR/VEGFR inhibitor, AEE788, in combination with RAD001 greatly decreased tumour growth in glioma xenografts (Goudar *et al*, 2005). Furthermore, targeting the glycolytic pathway in combination with mTOR inhibition may also be useful in cases where DNA-damaging agents are less efficient in inhibiting growth and promoting apoptosis of cancer cells (Xu *et al*, 2005).

## TARGETING THE TRANSLATIONAL APPARATUS AS A THERAPEUTIC APPROACH

A critical outcome of mTOR activation is the phosphorylation of several components of the translational apparatus, which mediate translation initiation and cell proliferation. A major rate-limiting step in translation initiation is the formation of the eIF4F complex at the 5′ mRNA cap structure. Rapamycin inhibits eIF4F formation largely in part through 4E-BP dephosphorylation. Several studies have implicated the eIF4E/4E-BP pathway as a putative downstream target of mTOR in tumorigenesis (Avdulov *et al*, 2004; Ruggero *et al*, 2004; Wendel *et al*, 2004). Inhibition of mTOR-mediated cap-dependent translation, through the use of rapamycin is insufficient to elicit a complete inhibition of cancer cell proliferation. This may be in part due to chemoresistance that arises as a consequence of Akt-mediated activation. Hence, the benefits of rapamycin treatment may not be realised in all cases of aberrant mTOR activity. Since experimental models implicate translation initiation directly in cancer cell growth, it would be of interest to develop molecules that specifically target downstream components of the mTOR signaling pathways, which control eIF4F formation.

Recent work has implicated microRNAs (miRNAs) in the regulation of translation initiation by inhibiting cap-dependent translation (Pillai *et al*, 2005). Importantly, many miRNAs function as oncogenes or tumour suppressors (Chen, 2005). Thus, it is possible that the mTOR pathway could control the activity of miRNAs in cancer. It will be important to continue to study the PI3K/Akt/mTOR pathway and its downstream effectors of translation as molecular targets for anticancer therapies.

## Figures and Tables

**Figure 1:**
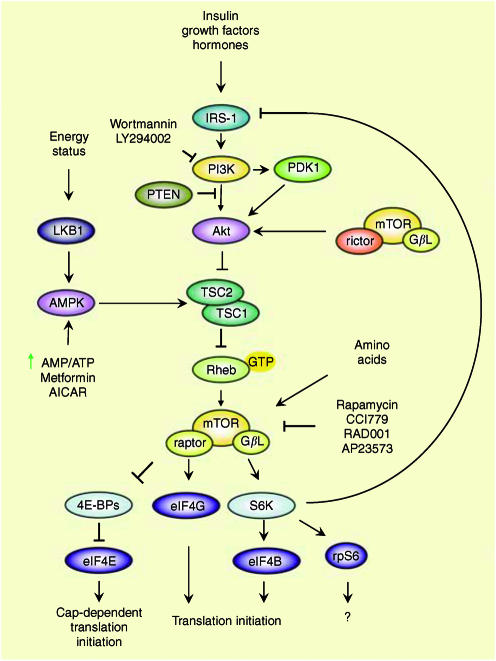
mTOR signaling to translation initiation. mTOR signaling regulates translation initiation by integrating several different inputs. Insulin, hormones and growth factors activate the PI3K/Akt signaling cascade. Akt receives inputs from PDK1 and the rictor/G*β*L/mTOR complex. Energy status (the AMP/ATP ratio) modulates AMPK activity. These pathways affect TSC1/2 to regulate mTOR activity via Rheb. The raptor/G*β*L/mTOR complex mediates the phosphorylation of 4E-BP and S6K. Pharmacological inhibition of PI3K (wortmannin or LY294002) and mTOR (rapamycin or its analogs), or activation of AMPK (by increased AMP/ATP, metformin or AICAR) are indicated.
